# Structural Changes and Mechanical Resistance of Claws and Denticles in Coconut Crabs of Different Sizes

**DOI:** 10.3390/biology10121304

**Published:** 2021-12-09

**Authors:** Tadanobu Inoue, Shin-ichiro Oka, Koji Nakazato, Toru Hara

**Affiliations:** 1 National Institute for Materials Science, 1-2-1, Sengen, Tsukuba 305-0047, Japan; NAKAZATO.Koji@nims.go.jp (K.N.); HARA.Toru@nims.go.jp (T.H.); 2 Okinawa Churashima Foundation, 888 Ishikawa, Motobu, Okinawa 905-0206, Japan; sh-oka@okichura.jp

**Keywords:** biomineralization, coconut crab, cuticle, denticle, nanoindentation, microstructure

## Abstract

**Simple Summary:**

Understanding the diverse mechanisms by which organisms achieve exceptionally high mechanical properties may enable the development of unique, biologically inspired materials. We assessed the microstructure, composition, and mechanical resistance of the pinching side with denticles and of the outer side without denticles in robust claws of coconut crabs with body weight (*BW*) of 300 g to 1650 g. Surprisingly, they were independent of *BW* except for low hardness near the surface of the denticles of a small crab of 300 g. Although the microstructure of the denticles was clearly different from that of the exocuticle, their mechanical resistance indicated the same maximum value. The denticle can be regarded as a bulge of the cuticle without phosphorus. The design principles found in the exoskeleton provided promising opportunities for the research and development of novel structural materials.

**Abstract:**

The exoskeleton of the pinching side of claws with denticles and of the outer side without them on the coconut crab, *Birgus latro*, which is a rare organism, were studied using a materials science approach. The mechanical resistance of three claws of different sizes was investigated along the exoskeleton thickness from the outer surface to the inner surface, and the results were compared, including the contribution of the microstructure and chemical compositions. Mechanical properties, hardness (*H*) and stiffness (*E_r_*), were probed through nanoindentation tests. The results showed the *H*, *E_r_*, microstructures, and chemical components of the exocuticle and endocuticle layers were almost the same, in a BW range of 300 g to 1650 g. At the same time, the *H* and *E_r_* near the surface of the denticles of a small coconut crab of 300 g were lower than those of other large coconut crabs. The microstructure of the denticles was clearly different from that of the exocuticle, but the maximum mechanical properties near their surface indicated almost the same values, *H_max_* = 4 GPa and *E_r_*_(*max*)_ = 70 GPa, regardless of being on the pinching side or the outer side. A denticle can be regarded as a bulge of the cuticle without phosphorus and with high magnesium. The results provided novel information that expanded our knowledge about the claw microstructure of coconut crabs with different body sizes, and may be used in further studies

## 1. Introduction

Organisms have a tissue structure that is adaptive to the environment. Understanding the diverse mechanisms by which organisms achieve exceptionally high mechanical properties may enable the development of unique, biologically inspired materials [1,2,3,4,5,6,7,8]. In fact, bio-inspired materials with excellent functional and mechanical properties have been reported [9,10,11,12,13,14]. The exoskeleton that covers the outside of the arthropod body is very hard, and its hardness is associated with calcification and tissue structure [15,16,17,18,19]. The tissue structure in a mineralized cuticle is characterized by a twisted plywood pattern [20,21,22,23,24,25]. In recent studies, it was reported that the complicated structure of this pattern was characterized with 3D analysis [22], and the hardness increased as the stacking height of the twisted plywood structure decreased [25]. Wu et al. [26] showed structural design strategies for the optimized effective fracture energy depending on the pitch angles, fiber lengths, fracture energy of crack bridging, and twist angle distribution in composites with this plywood pattern structure. Furthermore, the fabrication techniques and the corresponding mechanical properties of Bouligand biomimetic cellulose nanocrystal nanocomposites have been reported [27]. Since the Bouligand structure is the additional complexity involved in creating a helical twist in the layer-by-layer stacking, it may have thus far received less attention. However, new technologies such as 3D analysis and 3D and 4D printing [28,29] have made it possible to manufacture complex structures. A detailed understanding of the complex tissue structure in organisms is becoming more important.

The exoskeleton of crustaceans protects the organisms from predatory attacks and water loss. Many decapod crustaceans have characteristically large claws that are used to capture prey, crush a wide variety of rigid foods, and protect themselves from predators. Furthermore, in some species, the claws are used in mating, and their presence/absence may be crucial in mating success [30,31]. Hence, the claws have superior mechanical properties and strong calcification as compared to the carapace and legs [18,25,32,33,34].

**Figure 1:**
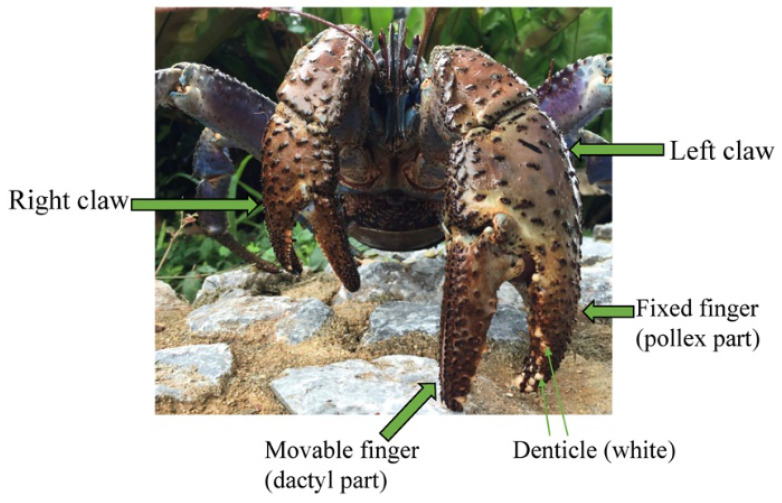
Photograph of coconut crab with robust/mighty claws.

The coconut crab, *Birgus latro*, [35] has an extraordinary pinching force as compared to other decapod crustaceans. Surprisingly, it has a pinching force of more than 90 times its body weight [36], and exerts the strongest force of almost all terrestrial animals with body weight near 1 kg [22]. Usually, the left claw of the coconut crab is larger than the right claw. The claw is composed of two articulating parts: the dactyl part is movable (often referred to as the “movable finger”), and the pollex part is fixed (the “fixed finger”), as shown in Figure 1. The fixed finger is the side that supports the force when pinched and is important for maintaining the function of the claw. In the claw fingers, there is an inner part that pinches prey (pinching side) and an outer part (outer side). The pinching side displays toothlike denticles, which come into direct contact with predators and prey. The denticles of the fixed finger in general are larger than these of the movable finger. These denticles have little or no epi- or exocuticle in this region in various crabs: *Cancer borealis*, *Callinectes sapidus*, *Chionoecetes opilio*, *Paralomis birsteini*, *Paralithodes camtschaticus*, and *Scylla serrata* [37,38]. Nevertheless, the hardness was 2.5 to 10 times higher in the denticle than in the endocuticle. The denticles serve as the points of contact in the capture of prey and defense against predators. Therefore, the denticles are expected to be abrasion-resistant, and may have excellent mechanical properties in the claw. Furthermore, these denticles are present in various sizes and are irregularly arranged [38], as shown in Figure 1, and these characteristics could possibly change with growth. However, there are few studies on the tissue and mechanical properties of denticles in the exoskeleton. Here, we prepared three left claws of the coconut crabs with different body weights and quantified their hardness, stiffness, tissue structure, and chemical compositions along the exoskeleton thickness from the outer surface to the inner surface in the claw cross section of the pinching side with the denticles of each fixed finger and the outer side without them.

## 2. Materials and Methods

### 2.1. Specimen Preparation

**Figure 2:**
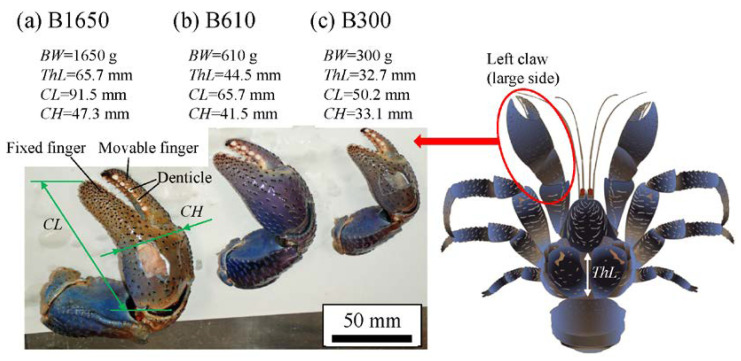
Photographs of the left claws of coconut crabs with different body weights of (**a**) 1650 g, (**b**) 610 g, and (**c**) 300 g used in the present study. Here, *BW* denotes the body weight, *ThL* the thoracic length, *CL* the claw length, and *CH* the claw height.

Coconut crabs were captured at Motobu town on northern Okinawa Island, located in southwestern Japan. As part of an ecological survey of wild coconut crabs inhabiting this area, their body weight (*BW*) and size (*ThL*: thoracic length; *CL*: claw length; *CH*: claw height) were recorded, and only the left claw was collected for the sample. Here, the three claws shown in Figure 2 were prepared as follows. All three coconut crabs were male, and their *BWs* were 1650 g (B1650 specimen), 610 g (B610 specimen), and 300 g (B300 specimen). The claws were frozen, transported from Okinawa Churashima Foundation to National Institute for Materials Science in Tsukuba for analysis, and stored at −18 ℃ prior to analysis. The claws were thawed under running water, and then the fixed finger was cut using a saw. The fixed fingers were cut to fit within the dimensions of a 40 mm diameter mounting cup used in the embedding process. Embedding cups were filled with epoxy (Struers, EPOFIX resin), and the three specimens were left to cure at room temperature for approximately 12 h. The specimens were placed under vacuum for 10 min immediately after the addition of epoxy to further ensure penetration of the epoxy into the specimen voids. Then the specimens were ground with 320/600/800 grit SiC papers; subsequently polished with a 9, 3, and 1 μm diamond suspension; and finally polished with a 0.05 μm alumina suspension. After polishing, cross-sectional micrographs of the three specimens were obtained using an optical microscope (OM). Subsequently, the specimens were coated with about 2 nm of osmium in order to characterize the microstructure and chemical compositions. After this was completed, the specimens were repolished in order to remove osmium coating and subjected to nanoindentation tests. The specimen thickness shaved by repolishing was approximately 0.3 mm.

### 2.2. Microstructure Observation

For the microstructure characterization, a focused ion beam (FIB)–scanning electron microscope (SEM) dual-beam instrument (Thermo Fisher Scientific, Scios2, Waltham, Massachusetts, U.S.) was used. The typical SEM observation condition was an accelerating voltage of 2 kV and a secondary electron detector in a chamber or an in-lens type annular back-scattered electron detector. An energy-dispersive X-ray spectroscope (EDS) attached to this FIB-SEM was applied for the compositional analysis. A large silicon-drift-type detector (Oxford Instruments Ultim Max 170, Abingdon, Oxfordshire, G.B.) ensured high detecting efficiency and low statistical error in the quantitative analysis. An accelerating voltage of 15 kV was used when the EDS analysis was carried out. Before SEM observation, an osmium (Os) coating was treated on the sample surface of all specimens. The principle of the coater we used (Meiwafosis Co., Ltd., Tokyo, Japan) was based on a plasma chemical vapor deposition [39]. Os coating can add electron conductivity to eliminate electron charge-up for nonconductive organisms. By applying this method, we could obtain a clear and high-resolution image; in addition, since the thickness of the coated film was very thin, around 2 nm, the effect on the EDS compositional analysis was negligible.

### 2.3. Nanoindentation Tests

Nanoindentation testing was performed at ambient temperature using an Elionix ENT-NEXUS. The specimens for the dehydrated condition were placed in air for more than 48 h before indentation. The tests were conducted along an inner surface from an outer surface of the specimens with a Berkovich-type diamond indenter with an angle of 115° at a maximum force of 5 mN. The loading curve consisted of a 5 s loading time, holding for 5 s at the maximum force, and then a 5 s unloading time. The hardness (*H*) and reduced elastic modulus (*E_r_*) were analyzed from the unloading curve using the Oliver–Pharr method. Here, *E_r_* was employed as the stiffness as in pioneering studies on biological research [15,24,40,41,42].

## 3. Results

### 3.1. Cross-Sectional Images

**Figure 3:**
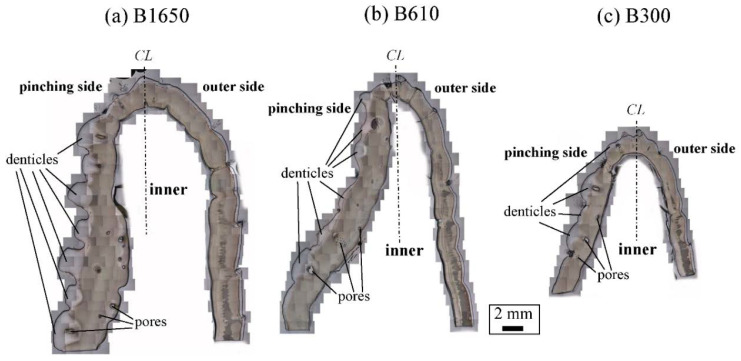
Optical micrographs of a cross section of the fixed finger of (**a**) B1650, (**b**) B610, and (**c**) B300 after polishing.

**Figure 4:**
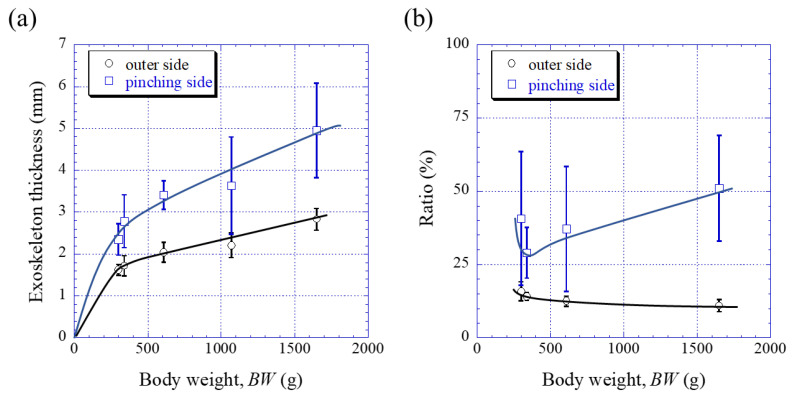
Variations in (**a**) the exoskeleton thickness on the outer side and on the pinching side with body weight (*BW*) and in (**b**) the ratio (=exocuticle thickness/exoskeleton thickness) on the outer side, as well as the ratio (=denticle thickness/exoskeleton thickness) on the pinching side with *BW*. Here, error bars represent standard deviations. Data were based on the measurement results shown in Figure S2 in the Supplementary Materials.

Figure 3 shows optical micrographs of a cross section of the fixed finger. In these micrographs, the pinching side with the denticles is on the left, and the outer side without denticles is on the right. On the outer side, the thickness of the exoskeleton was not perfectly constant, and narrow areas were observed in some places. As can be seen in Figure S1 in the Supplementary Materials, these narrow areas were associated with sensory hairs formed from the inner side through relatively thick pore canals. The sensory hairs could also be observed on the claws shown in Figure 2, and were mainly found on claws and legs. Interestingly, in Figure 3 and Figure S1, some pores were observed on the pinching side, and these pores were located within the denticles or within the endocuticle layer below the denticles. The exocuticle layer, endocuticle layer, and denticles were clearly observed in all specimens. The results of measuring the ratio of the exocuticle thickness to the exoskeleton thickness on the outer side, as well as the ratio of the denticle thickness to the exoskeleton thickness on the pinching side, are displayed in Figure S2 in the Supplementary Materials. The results are summarized in Figure 4, in which data for the male coconut crabs (340 g and 1070 g) inhabiting northern Okinawa Island we have measured so far are included.

### 3.2. Hardness and Stiffness

**Figure 5:**
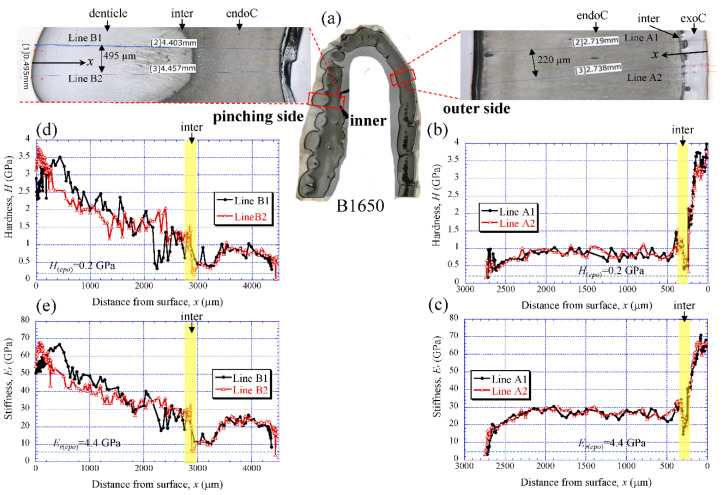
(**a**) Areas where nanoindentation tests were conducted in the B1650 specimen. Distributions of (**b**) hardness, *H*, and (**c**) stiffness, *E_r_*, with distance from the surface, *x*, on the outer side; and of (**d**) *H* and (**e**) *E_r_* with *x* on the pinching side. Here, *H*_(*epo*)_ and *E_r(epo)_* denote the hardness and stiffness of cold epoxy resin, respectively.

Figure 5a shows the areas of the nanoindentation measurement of the B1650 specimen, and Figure 5b–e show the lateral distribution of mechanical resistance: the hardness (*H*) and the reduced stiffness (*E_r_*). Note that *x* refers to the distance from the outer surface of each side. Both the *H* and the *E_r_* revealed pronounced gradients between the surface and the inner side. On the outer side (Figure 5b,c), the *H* was the range of 3 to 4 GPa in the exocuticle layer, and decreased abruptly toward the intermediate layer. The value was 0.3–0.5 GPa. Subsequently, the *H* increased to 1.5 GPa and was in the range of 0.7 to 1.0 GPa in the endocuticle layer. The *E_r_* also showed the same change through the exoskeleton thickness. The value was in the range of 60 to 70 GPa in the exocuticle layer, decreased to 14–18 GPa in the intermediate layer and subsequently increased to 34 GPa, and was 22–30 GPa in the endocuticle layer. On the other hand, on the pinching side with the denticles (Figure 5d,e), although the two lines (B1 and B2) showed slightly different changes near the surface, the distributions of the *H* and the *E_r_* showed basically the same trends as the distributions on the outer side. They gradually decreased in the region of the denticle, decreased abruptly in the intermediate layer and subsequently increased, and become constant in the exocuticle layer. Surprisingly, the minimum values in the intermediate layer corresponded to the mechanical resistance of the cold epoxy resin used as the embedding material. These characteristics of mechanical resistance seen in the B1650 specimen were also obtained in the B610 and the B300 specimens.

**Figure 6:**
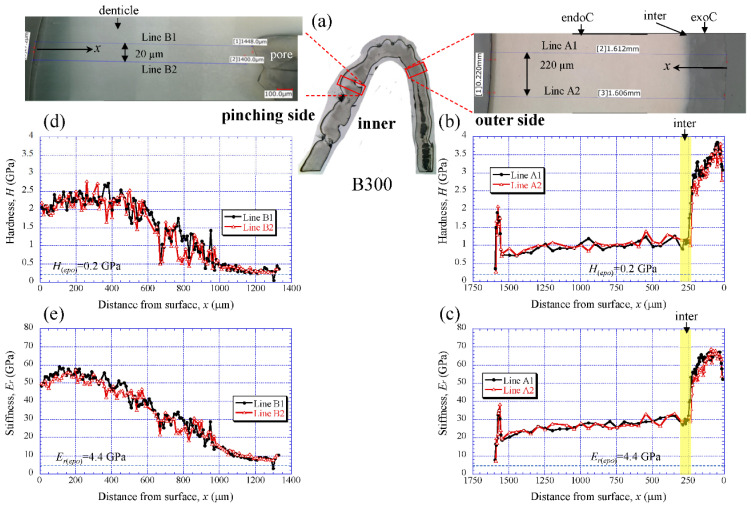
(**a**) Areas where nanoindentation tests were conducted in the B300 specimen. Distributions of (**b**) hardness, *H*, and (**c**) stiffness, *E_r_*, with distance from the surface, *x*, on the outer side; and of (**d**) *H* and (**e**) *E_r_* with *x* on the pinching side. Here, *H*_(*epo*)_ and *E_r(epo)_* denote the hardness and stiffness of cold epoxy resin, respectively.

Figure 6 shows the results for the B300 specimen. On the outer side (Figure 6b,c), the *H* steadily increased from the surface, and the peak value occurred at about 50 µm in the exocuticle layer. Subsequently, it gradually decreased, decreased abruptly in the intermediate layer, and then slightly decreased in the endocuticle layer. The *E_r_* also exhibited the same distributions as the *H*. The maximum values of *H* and *E_r_* in the exocuticle layer and the values in the endocuticle layer were almost the same as in the B1650 specimen shown in Figure 5b,c. The *H* and *E_r_* of the pinching side shown in Figure 6d,e steadily increased from the surface and then gradually decrease in the denticles. The *H* and *E_r_* of near the surface of the denticle in B300 were found to be smaller than those in B1650, as shown in Figure 5d,e.

**Figure 7:**
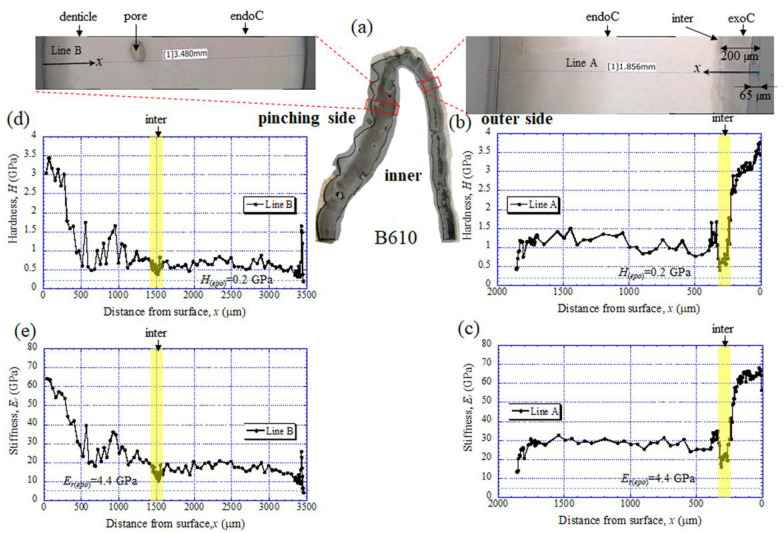
(**a**) Areas where nanoindentation tests were conducted in the B610 specimen. Distributions of (**b**) hardness, *H*, and (**c**) stiffness, *E_r_*, with distance from the surface, *x*, on the outer side; and of (**d**) *H* and (**e**) *E_r_* with *x* on the pinching side. Here, *H*_(*epo*)_ and *E_r(epo)_* denote the hardness and stiffness of cold epoxy resin, respectively.

**Figure 8:**
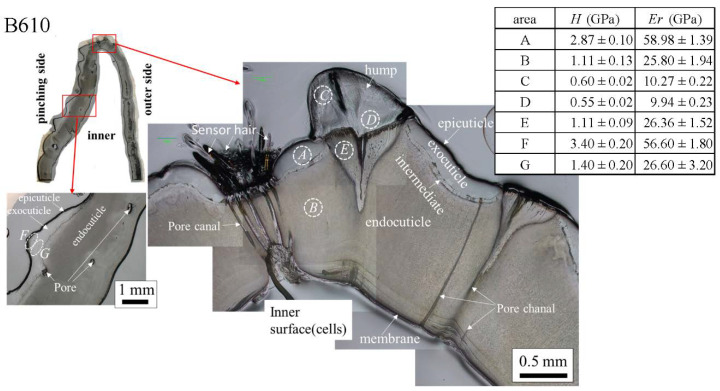
Hardness, *H*, and stiffness, *E_r_*, at seven characteristic areas (A to G) in the B610 specimen obtained from nanoindentation tests. The tests were conducted five times for each area.

The results for the B610 specimen are shown in Figure 7 and Figure 8. In this specimen, only one line was measured on each side, and instead a nanoindentation test was also performed at seven characteristic areas, from A to G. These areas of the claw tip were similarly observed in the other two specimens, as shown in Figure 3, Figures S1 and S2. The distributions and values shown in Figure 7b–e were fairly close to those of the B1650 specimen shown in Figure 5b–e. Figure 8 shows the results at seven areas, from A to G. A and B corresponded to the center areas in the exocuticle and endocuticle layers, respectively. C and D were the areas of the hump observed in Figure 8; the humps were seen near the top in all specimens, and they corresponded to the many black protrusions seen in the claw surface in Figure 1 and Figure 2. E was the area of distinctly different tissues below the hump. F and G on the pinching side were areas near the surface of the denticle and at the center of the denticle thickness. The *H* and *E_r_* in areas A and B and in areas F and G were consistent with the results for the outer side and the pinching side shown in Figure 7. The *H* and *E_r_* in area C were the same as in area D; these values were much smaller than in areas F and G in the denticle and area B in the endocuticle. That is, the hump was not the denticle. The many black protrusions seen on the claw surface and the claw top in coconut crabs may have been keratin or chitin, a nonmineralized area [43]. This point will be examined through detailed tissue analysis and component analysis in the future. The values in area E corresponded to those in area B.

### 3.3. Chemical Compositions

**Figure 9:**
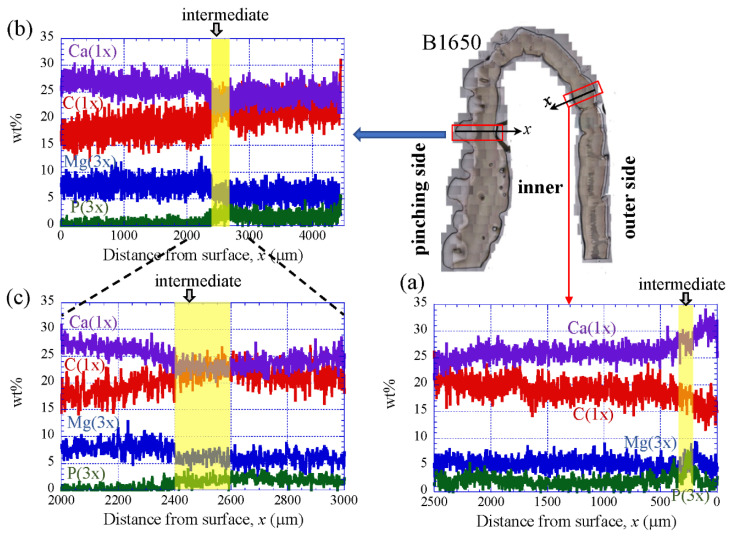
Line-scanning profiles with distance from the surface of the B1650 claw cross-sectional plane measured by energy-dispersive X-ray spectroscopy (EDS): (**a**) outer side; (**b**,**c**) pinching side. Here, the weight percentages of magnesium (Mg) and phosphorus (P) were expanded by a factor of 3 relative to those of calcium (Ca) and carbon (C).

**Figure 10:**
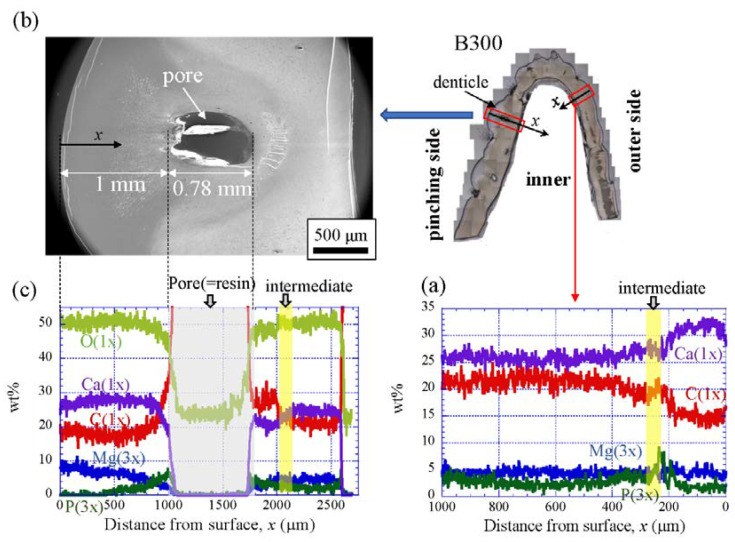
Line-scanning profiles with distance from the surface of the B300 claw cross-sectional plane measured by EDS: (**a**) outer side; (**b**) SEM micrographs of the denticle on the pinching side; (**c**) line-scanning profiles of its area. Here, the weight percentages of magnesium (Mg) and phosphorus (P) were expanded by a factor of 3 relative to those of calcium (Ca), carbon (C), and oxygen (O).

**Figure 11:**
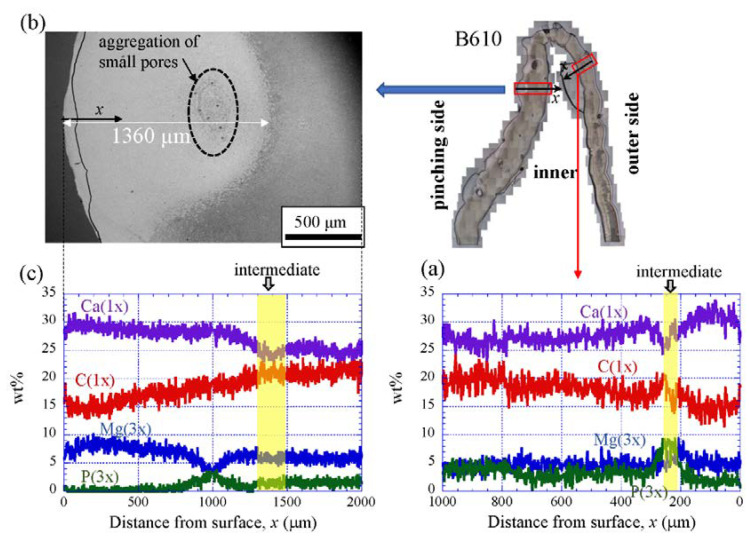
Line-scanning profiles with distance from the surface of the B610 claw cross-sectional plane measured by EDS: (**a**) outer side; (**b**) SEM micrographs of the denticle on the pinching side; (**c**) line-scanning profiles of its area. Here, the weight percentages of magnesium (Mg) and phosphorus (P) were expanded by a factor of 3 relative to those of calcium (Ca), carbon (C), and oxygen (O).

Figure 9 shows the line-scanning profiles of calcium (Ca), magnesium (Mg), phosphorus (P), and carbon (C) through the thickness of the outer side and pinching side in the B1650 specimen measured by EDS. Note that the weight % of Mg and P were expanded by a factor of 3 relative to those of Ca and C. The line scans revealed decreasing Ca concentrations from the surface to the specimen interior, regardless of which side, and a concomitant increase in C throughout the same region. Mg concentrations decreased slightly in the intermediate layer, while P concentrations increased. In the denticle, Ca concentrations were slightly lower than those in the exocuticle on the outer side, P concentrations were almost zero, and Mg concentrations were high as compared to the exocuticle and endocuticle. Mg concentrations in the endocuticle on the pinching side were the same as those in the endocuticle on the outer side. Similar trends were seen in the B300 specimen (Figure 10) and the B610 specimen (Figure 11). On the pinching side of the B300 specimen, since a large pore existed in the exoskeleton thickness, the inorganic components of Ca, Mg, and P became zero, and the organic components of C and O changed significantly in that region due to the effect of resin. The P concentration increased as it approached to the pore, while the Mg concentration decreased. These changes related to P and Mg were also seen in aggregations of small pores in the denticle of the B610 specimen, as shown in Figure 11. In all specimens, the Ca concentration in the exocuticle on the outer side was in the range of 28 to 33 wt %, and that in the denticle on the pinching side was in the range of 24 to 28 wt % regardless of *BW*. In short, no calcification in the exocuticle and denticle was strongly changed by *BW*.

### 3.4. Microstructures

**Figure 12:**
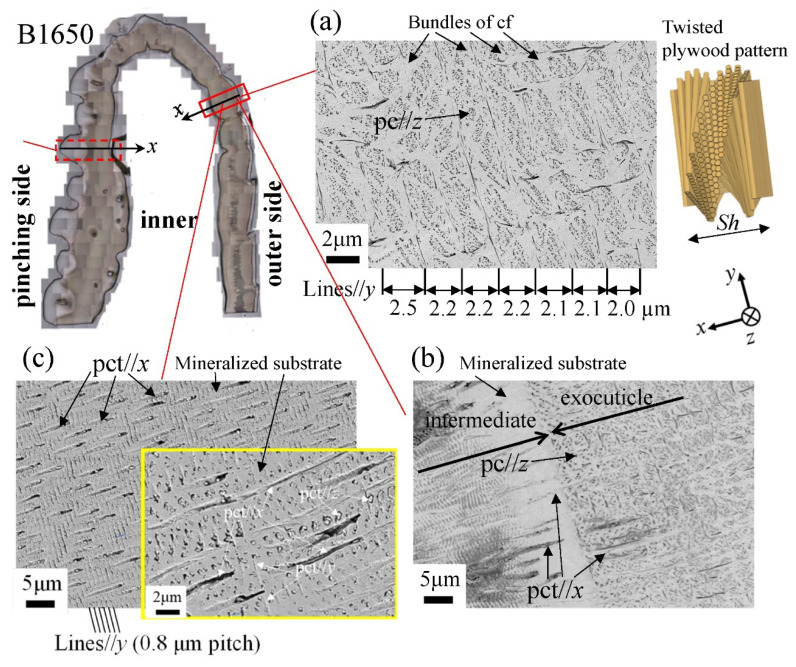
Scanning electron microscope (SEM) micrographs at (**a**) *x* = 110 µm (the exocuticle layer), (**b**) *x* = 240 µm (the intermediate layer), and (**c**) *x* = 330 µm (the endocuticle layer) on the outer side of the B1650 specimen. Here, cf denotes chitin fibers in the mineral–protein matrix, pct denotes pore canal tubules, and pc denotes pore canals.

**Figure 13:**
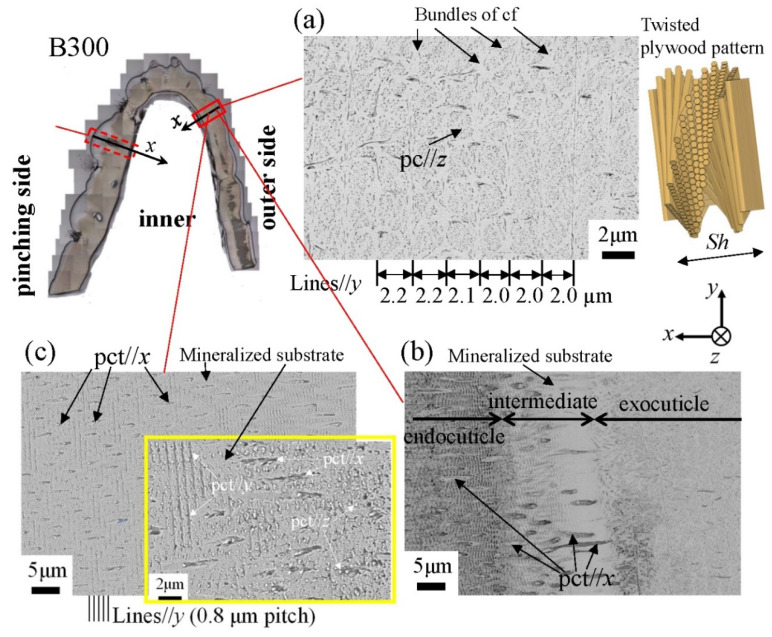
SEM micrographs at (**a**) *x* = 110 µm (the exocuticle layer), (**b**) *x* = 210 µm (the intermediate layer), and (**c**) *x* = 250 µm (the endocuticle layer) on the outer side of the B300 specimen. Here, cf denotes chitin fibers in the mineral–protein matrix, pct denotes pore canal tubules, and pc denotes pore canals.

Figure 12 shows SEM micrographs at three layers on the outer side of the B1650 specimen. The tissue structure remarkably exhibited a morphological change in the intermediate layer. In the exocuticle layer, the lamella thickness (Lines//*y*) observed in Figure 12a corresponded to the stacking height, *Sh*, in a twisted-plywood-patterned structure rotated 180° around an axis normal to the surface, which was characteristic of the cuticle of arthropods [15,20,22]. The *Sh* tended to decrease as it approached the surface [25]. On the other hand, the endocuticle layer shown in Figure 12c was a tissue structure in which thick pore canal tubules (pct//*x*) parallel to the *x*-direction and thin pct (pct//*y*, pct//*z*) in two directions normal to it intersected in the mineralized matrix [22]. The tissue structure in the outer side was the same as that in the other two specimens: the B300 specimen (Figure 13) and the B610 specimen. This was consistent with the results of a coconut crab of 1070 g reported earlier [22,25].

**Figure 14:**
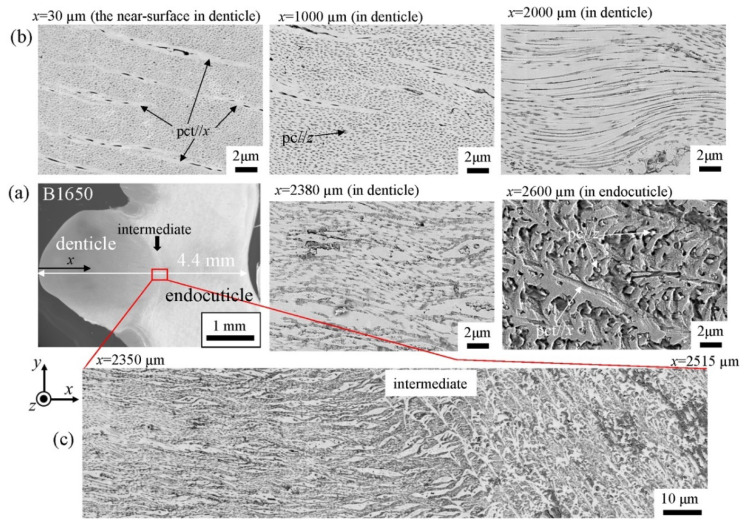
SEM micrographs (**a**) from the denticle to the endocuticle on the pinching side of the B1650 specimen, (**b**) at the distance from the surface, *x*, and (**c**) near the intermediate layer.

**Figure 15:**
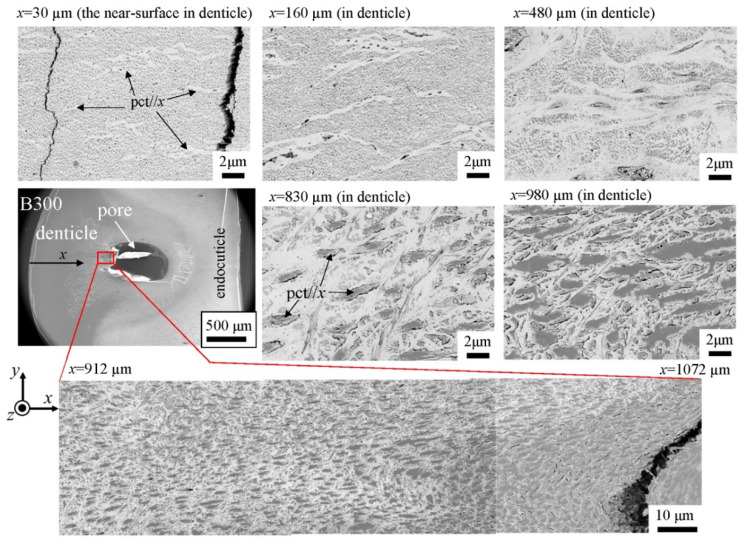
SEM micrographs from the denticle to the endocuticle on the pinching side of the B300 specimen.

**Figure 16:**
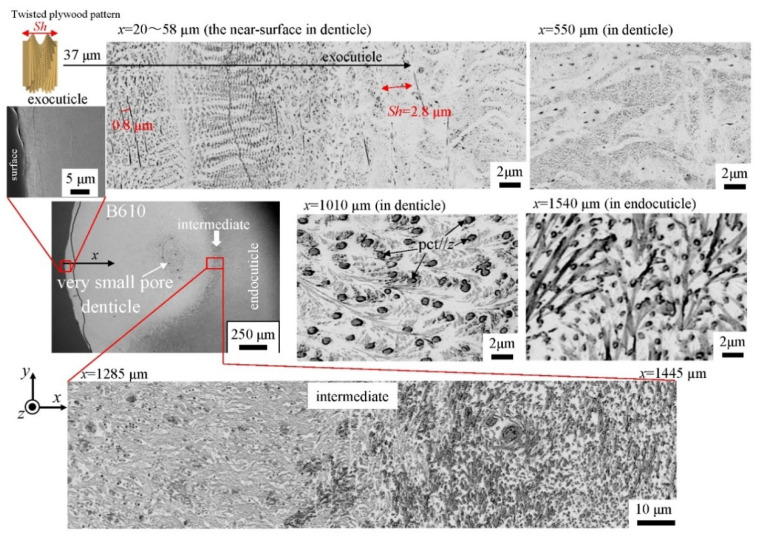
SEM micrographs from the denticle to the endocuticle on the pinching side of the B610 specimen.

**Figure 17:**
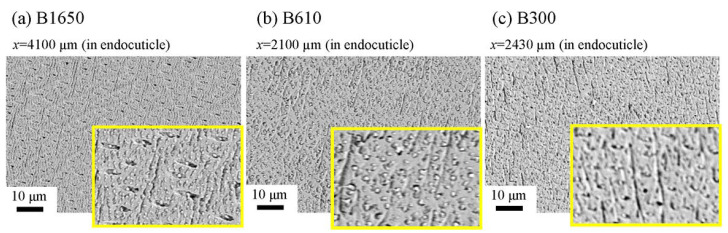
SEM micrographs of the endocuticle layer on the pinching side of (**a**) B1650, (**b**) B610, and (**c**) B300 specimens.

The microstructures on the pinching side of the B1650 specimen are shown in Figure 14. The tissue structure in the denticle was clearly different from that on the outer side. The white streaks (perhaps pct//*x*) parallel to the *x*-direction became wavy at *x* = 2000 µm and their density increased, while very small black dots parallel to the *z*-direction, pc//*z*, became larger. The low-magnification SEM (×3500) micrograph near the intermediate layer shown in Figure 14c revealed a distinct shift in tissue structure from the denticle to the endocuticle; i.e., the tissue flow changed from “parallel to the *x*-direction” to “parallel to the *y*-direction” in the intermediate layer. This meant that the tissue structure mainly aligned normally to the surface within the denticle. Figure 15 shows the microstructures on the pinching side in the B300 specimen. The tissue changes in the denticle exhibited basically the same characteristics. The denticle microstructures in the B610 specimen are shown in Figure 16. Curiously, a small amount of exocuticle layer was visible near the surface of the denticle. Its thickness covering the denticle was about 37 μm, forming a twisted-plywood-patterned structure of *Sh* ≈ 0.8–2.8 µm. This characteristic is also found in the red king crab, *Paralithodes camtschaticus* [37], of the same infraorder, Anomura, as the coconut crab. The microstructures within the endocuticle layer on the pinching side of all specimens are shown in Figure 17. The tissue structure corresponded to that in the endocuticle layer of the outer side.

## 4. Discussion

The body and claws of a coconut crab grow larger with age through molting [35]. The lifespan of coconut crab is said to be about 50 years, and its growth rate is very slow among crustaceans. In the case of the adult male coconut crab used in the present study, B300 was estimated to be 8–9 years old, B610 to be 11–13 years old, and B1650 to be 20–26 years old [44]. Adult coconut crabs of the Okinawa population molt only once per year, during the winter dray season. All crabs were obtained in July, with half a year passed since the last molting, so that the tissue structure, components, and thickness of the exoskeleton depended only on body size.

### 4.1. Change of Exoskeleton Thickness

In Figure 4a, the exoskeleton thickness regardless of the outer side and pinching side increased with increasing *BW*. This tendency was consistent with body size change, in that the *ThL* increased with *BW* [44]. Furthermore, the exoskeleton thickness on the pinching side with denticles (the blue line) was thicker than that on the outer side (the black line). This suggested that the presence of denticles on the pinching side is more important for claws. The size and arrangement of denticles that are not seen on the outer side of the claws are heterogeneous and are likely to change significantly during their growth. Hence, the standard deviation for the ratio of the denticle on the pinching side was very large, as shown in Figure 4b. On the outer side, even if the *BW* increased more than fivefold, the microstructures, chemical components, and mechanical resistance (*H* and *Er*) of the exocuticle and endocuticle layers were almost the same regardless of *BW*, as shown in Figure 5b,c, Figure 6b,c, Figure 7b,c, Figure 9a, Figure 10a, Figure 11a, Figure 12 and Figure 13. This meant that the robust exoskeleton of the claw on the coconut crab had already been formed at a *BW* of 300 g. However, the B300 specimen had the highest ratio of exocuticle in the exoskeleton, as shown by the black line in Figure 4b. This may have been because the function of the claw is maintained by increasing the ratio of the hard and rigid exocuticle layer until the exoskeleton becomes thicker by growing up.

### 4.2. Denticle Features on the Pinching Side

The denticles had different characteristics from the exocuticle. As shown in Figure 9b,c, Figure 10c and Figure 11c, in the denticle, the P concentration was almost zero, the Mg concentration was high, and the Ca concentration was slightly low as compared to the exocuticle on the outer side. Furthermore, on the basis of line profiles data shown in Figure 9c, Figure 10c and Figure 11c, the chemical compositions in the denticle did not change significantly with the *BW*. Curiously, there was a dense region of small pores in the lower portion of the denticle or in the endocuticle beyond the intermediate layer, where the P concentration was high and the Mg concentration was low (Figure 10b,c and Figure 11b,c). Furthermore, since the mechanical resistance in that region was low, a large pore of about 1 mm appeared as the polishing progressed. This region seems to be important as a place to supply necessary ions and nutrients from inner cells during the formation of the denticles [45]. The microstructure of the denticle flowed normally to the surface and changed from the surface to the inside, as shown in Figure 14, Figure 15 and Figure 16. This feature also can be visible in claw denticles of brachyuran crabs and anomuran crabs [37], and in mandible teeth of the crayfish *Cherax quadricarinatus* [46]. In connection with this change in tissue structure, the mechanical resistance gradually decreased from the surface to the inside, as shown in Figure 5d,e, Figure 6d,e and Figure 7d,e. Interestingly, the mechanical resistance near the surface of the denticle depended on the *BW*. The mechanical resistance (*H* = 2–2.5 GPa and *E_r_* = 50–60 GPa) at the surface of the denticle for the B300 specimen was lower than that for the B1650 and B610 specimens (*H* = 3–4 GPa and *E_r_* = 60–70 GPa). These maximum values for the B1650 and B610 specimens corresponded to those for the exocuticle on the outer side, which was independent of *BW*. The denticles, which exist only on the pinching side of the claw, are the place where the claw comes into direct contact with predators and prey, and a large force is locally applied to them when pinched. Based on the mechanics of materials, when a force is applied only to the denticle, the force is concentrated on a tip or a piece of the denticle, compared to when the force is applied on a flat surface without denticles. Although the denticle itself is very hard and fragile, when the prey is pinched in the region where many large and small denticles irregularly exist, the force applied to the claws can be effectively dispersed, and damage to the claws can be prevented. This effect is related to the size and number (distance) of stress-concentration parts such as denticles or cracks [13,47,48,49]. At the same time, large stress is locally generated in the place of predators and prey sides where the denticles come into direct contact. The size and number of denticles present on the pinching contact surface are as important a function of robust claws as the calcified exoskeleton. Surprisingly, the denticle tissue was arranged normal to the surface, as shown in Figure 14, Figure 15 and Figure 16. With this structure, cracks tend to progress inside, and the claws are more likely to be damaged. However, since the mechanical resistance in the intermediate layer between the denticle and the endocuticle is as soft and has a similar low stiffness as the resin, only the denticles are thought to be lost before the claws are fatally damaged when pinching. In fact, we sometimes see wild coconut crabs that have lost some denticles. Since new denticles grow from inside the exoskeleton after the denticles are lost, the size and arrangement of the denticles are heterogeneous and irregular (as seen in Figure S3 in the Supplementary Materials, in the photograph of the left claw of the coconut crabs with *BW* = 1070 g and 910 g, and Figure 1), and they depend on the individual coconut crab.

The tissue structure of the outer side of the claws is important not only for receiving the force of the claws, but also for protecting the body. The denticles on the pinching side exist for the specialized function of pinching prey like teeth. In other words, the structure on the pinching side of the claws is considered to have improved toughness due to the effect that disperses the force when pinching. The combination of extremely high pinching force and a highly resistant denticle results in greater ease of prey handling and a wider range of food items available for consumption. The low mechanical resistance of the denticles for the B300 specimen may have been associated with the pinching force of the coconut crab, which had a positive correlation with *BW*, and it should depend on the microstructure. The pinching force of each crab was estimated to be 1353 N for the B1650 specimen, 501 N for the B610 specimen, and 246 N for the B300 specimen [36]. The microstructure of the denticles was different from the microstructures of the exocuticle (twisted-plywood-pattern structure) and the endocuticle (porous structure) [22,25]. However, since the microstructure of the denticle was very complicated, it could not be perfectly characterized by SEM. We plan to clarify the denticle microstructure through three-dimensional analysis in the future.

### 4.3. Abrasion Resistance of the Denticle and Exo- and Endocuticle

**Figure 18:**
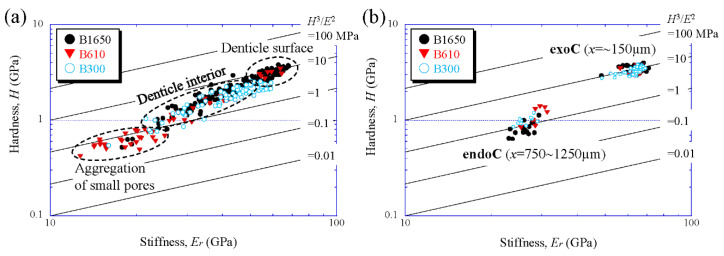
Property maps for abrasion resistance on (**a**) the denticle and (**b**) the exocuticle and endocuticle layers on the outer side for all specimens. Here, data lying on a straight line of *H*^3^/*E*^2^ indicated materials with equivalent performances in abrasion resistance. **exoC**: exocuticle layer; **endoC**: endocuticle layer.

To compare the mechanical properties of each layer of the coconut crab exoskeleton, the results were plotted on a map based on the abrasion resistance [50]. Figure 18 shows property maps with data obtained via nanoindentation tests for all specimens. Data were classified into the denticle, the exocuticle layer (*x* = surface~150 µm), and the endocuticle layer (*x* = 750–1250 µm). Here, data lying on a straight line of *H*^3^/*E*^2^ indicated materials with equivalent performances on the resistance of abrasion [51]. We found that the data were independent of *BW,* except near the tip of the denticle. The abrasion resistance near the tip of the denticle of the B300 specimen was lower than that of other large coconut crabs. This must be strongly related to the pinching force, which was proportional to the body weight of the coconut crab. Data for near the tip of the denticle of a crab weighing more than 610 g were in perfect agreement with those for the exocuticle layer. These data, *H_max_* = 4 GPa and *E_r_*_(*max*)_ = 70 GPa, were the limits of the local mechanical resistance of the coconut crab exoskeleton. Therefore, the denticle did not have particularly excellent abrasion resistance in the exoskeleton. The abrasion resistance of the denticle/exocuticle in the coconut crab was classified into materials with *H*^3^/*E*^2^ = 13. This property was higher than that in the data of all engineering polymers, and is comparable to data of the hardest metallic alloys, and even some of the softer ceramics [40]. In the denticle, except for the near-surface, the map revealed that the *H*–*E_r_* balance decreased from the surface to the inner part and approached the balance of the endocuticle layer. Furthermore, this feature was independent of *BW*. The denticle can be regarded as a bulge of the cuticle without phosphorus and with high magnesium. It has evolved as a function only on the pinching side of the claws to catch and crack prey and food.

## 5. Conclusions

The hardness, stiffness, tissue structure, and chemical compositions along the exoskeleton thickness of the pinching side with denticles and the outer side on the fixed finger of the left claws of the coconut crabs with different body weights (*BWs*) of 1650 g, 610 g, and 300 g were compared. The main results were as follows:

The microstructures, chemical components, and mechanical resistance (hardness, *H*, and stiffness, *E_r_*) of the exocuticle and endocuticle layers were almost the same in the *BWs* ranging from 300 g to 1650 g.

The exoskeleton of the pinching side with the denticles was thicker than that of the outer side without the denticles. The mechanical resistance and chemical composition in the exoskeleton thickness with denticles were the same regardless of *BW*, but the mechanical resistance of the denticles of the small coconut crab weighing 300 g was lower than that of large crabs.

The microstructure of the denticles was clearly different from that of the exocuticle and the calcium concentration in the denticles was slightly lower than that in the exocuticle. However, the maximum hardness and stiffness near their surface indicated almost the same values, *H_max_* = 4 GPa and *E_r_*_(*max*)_ = 70 GPa. The denticle can be regarded as a bulge of the cuticle without phosphorus and with high magnesium.

## Data Availability

Not applicable.

## Supplementary Materials

The following are available online at https://www.mdpi.com/article/10.3390/biology10121304/s1, Figure S1: Cross-sectional micrographs of the fixed finger in the claws of coconut crabs: (a) B1650, (b) B610, and (**c**) B300 after polishing. Some pores or aggregation of small pores were observed on the pinching side, and these pores were located within the denticles or within the endocuticle layer below the denticles. Figure S2: Optical micrographs of a cross section of the fixed finger of: (a) B1650, (b) B610, and (**c**) B300 after polishing. Figure S3: Heterogeneous/irregular denticles (white) of the fixed finger on the left claw of male coconut crabs: (a) body weight: 1070 g, thoracic length: 62 mm (S1); and (b) body weight: 910 g, thoracic length: 48.7 mm.
