# Supplementary material for: Structural Changes and Mechanical Resistance of Claws and Denticles in Coconut Crabs of Different Sizes
Source: Biology (Basel). 2021 Dec 9;10(12):1304. doi: 10.3390/biology10121304 (PMC8698411; doi:10.3390/biology10121304)
Supplement: Supplementary file 1 [file biology-10-01304-s001.zip › biology-1501048-supplementary.pdf]

biology MDPI

Supplementary Materials

Structural Changes and Mechanical Resistance of Claws and Denticles in Coconut Crabs of Different Sizes

Tadanobu Inoue, Shin-ichiro Oka, Koji Nakazato and Toru Hara

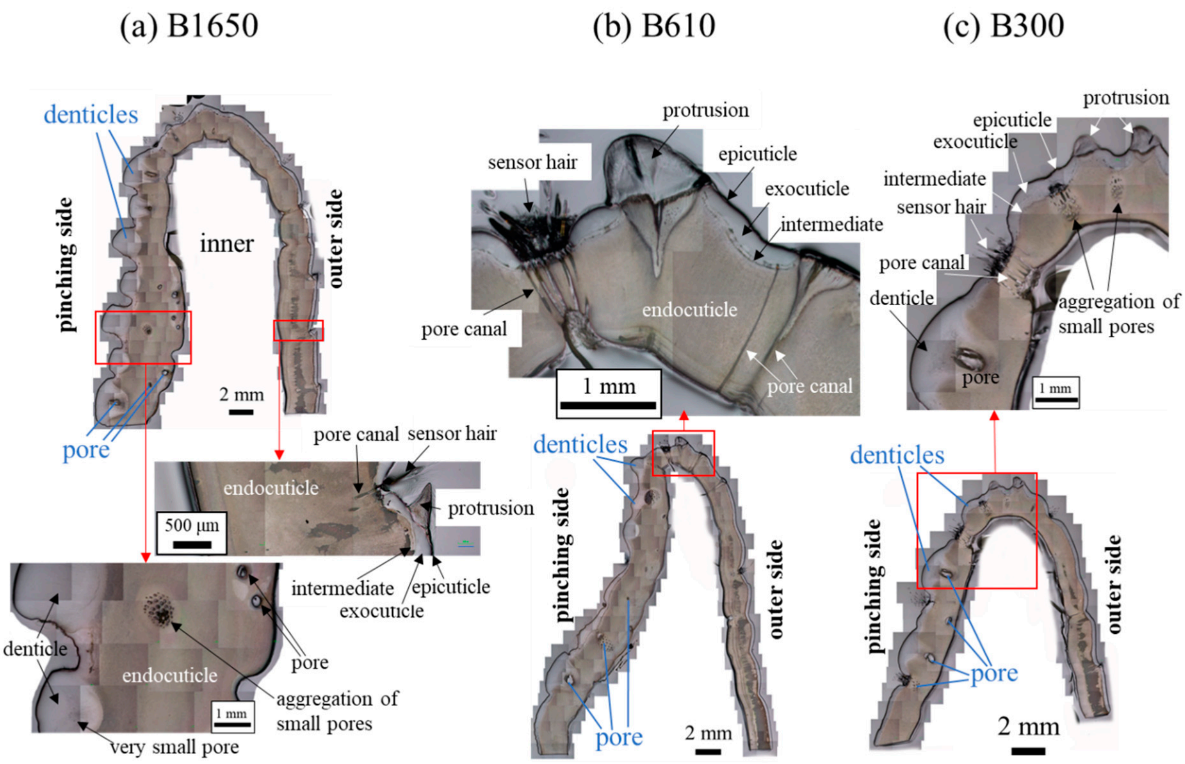

Figure S1. Cross-sectional micrographs of the fixed finger in the claws of coconut crabs (**a**) B1650, (**b**) B610, and (**c**) B300 after polishing. Some pores or aggregation of small pores were observed on the pinching side, and these pores were located within the denticles or within the endocuticle layer below the denticles.

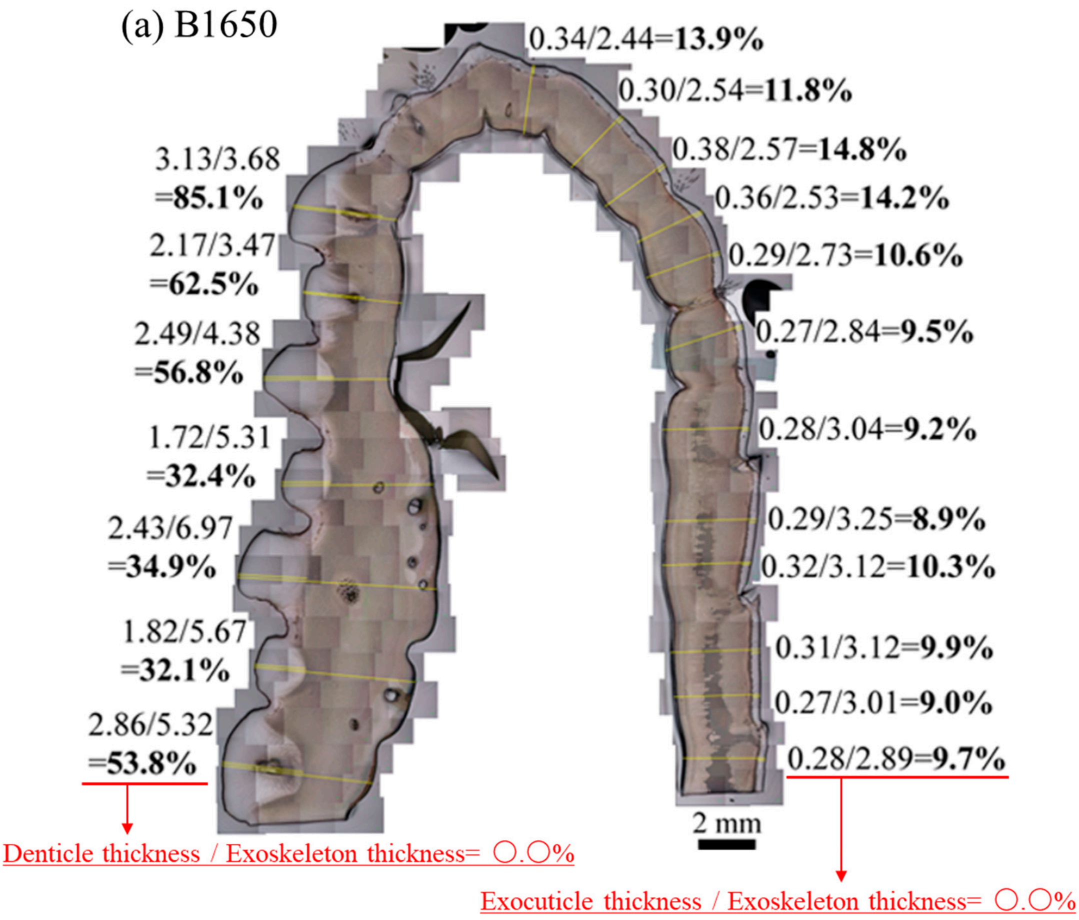
(a) B1650
0.34/2.44=13.9%
0.30/2.54=11.8%
0.38/2.57=14.8%
0.36/2.53=14.2%
0.29/2.73=10.6%
0.27/2.84=9.5%
0.28/3.04=9.2%
0.29/3.25=8.9%
0.32/3.12=10.3%
0.31/3.12=9.9%
0.27/3.01=9.0%
0.28/2.89=9.7%
3.13/3.68 =85.1%
2.17/3.47 =62.5%
2.49/4.38 =56.8%
1.72/5.31 =32.4%
2.43/6.97 =34.9%
1.82/5.67 =32.1%
2.86/5.32 =53.8%
2 mm
Denticle thickness / Exoskeleton thickness= ○.○%
Exocuticle thickness / Exoskeleton thickness= ○.○%

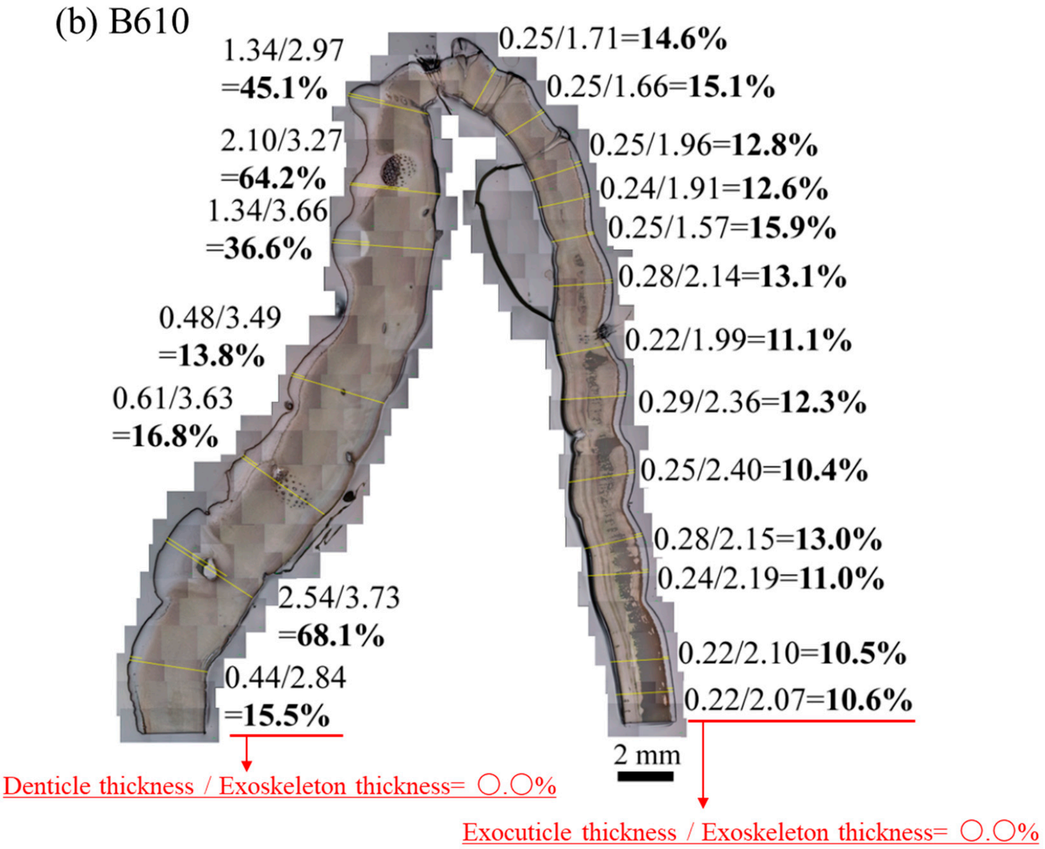
(b) B610
1.34/2.97 =45.1%
2.10/3.27 =64.2%
1.34/3.66 =36.6%
0.48/3.49 =13.8%
0.61/3.63 =16.8%
2.54/3.73 =68.1%
0.44/2.84 =15.5%
0.25/1.71=14.6%
0.25/1.66=15.1%
0.25/1.96=12.8%
0.24/1.91=12.6%
0.25/1.57=15.9%
0.28/2.14=13.1%
0.22/1.99=11.1%
0.29/2.36=12.3%
0.25/2.40=10.4%
0.28/2.15=13.0%
0.24/2.19=11.0%
0.22/2.10=10.5%
0.22/2.07=10.6%
2 mm
Denticle thickness / Exoskeleton thickness= ○.○%
Exocuticle thickness / Exoskeleton thickness= ○.○%

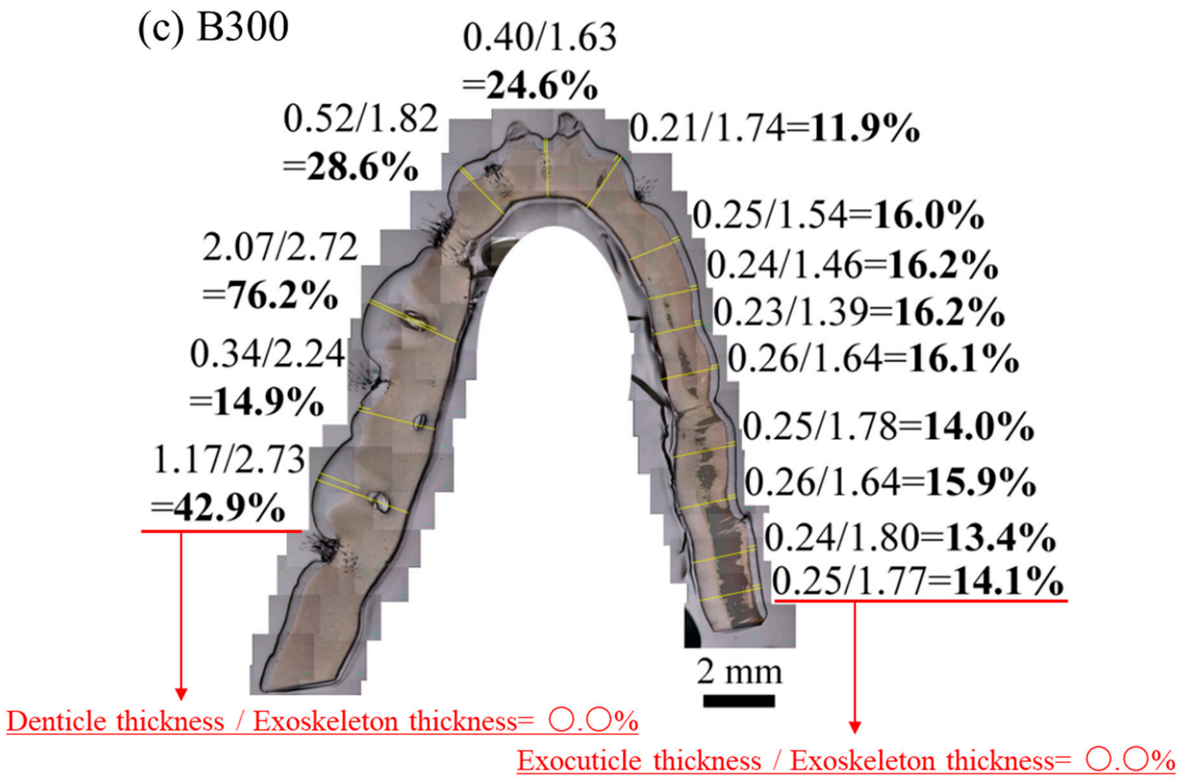

Figure S2. Optical micrographs of a cross section of the fixed finger of (**a**) B1650, (**b**) B610, and (**c**) B300 after polishing.

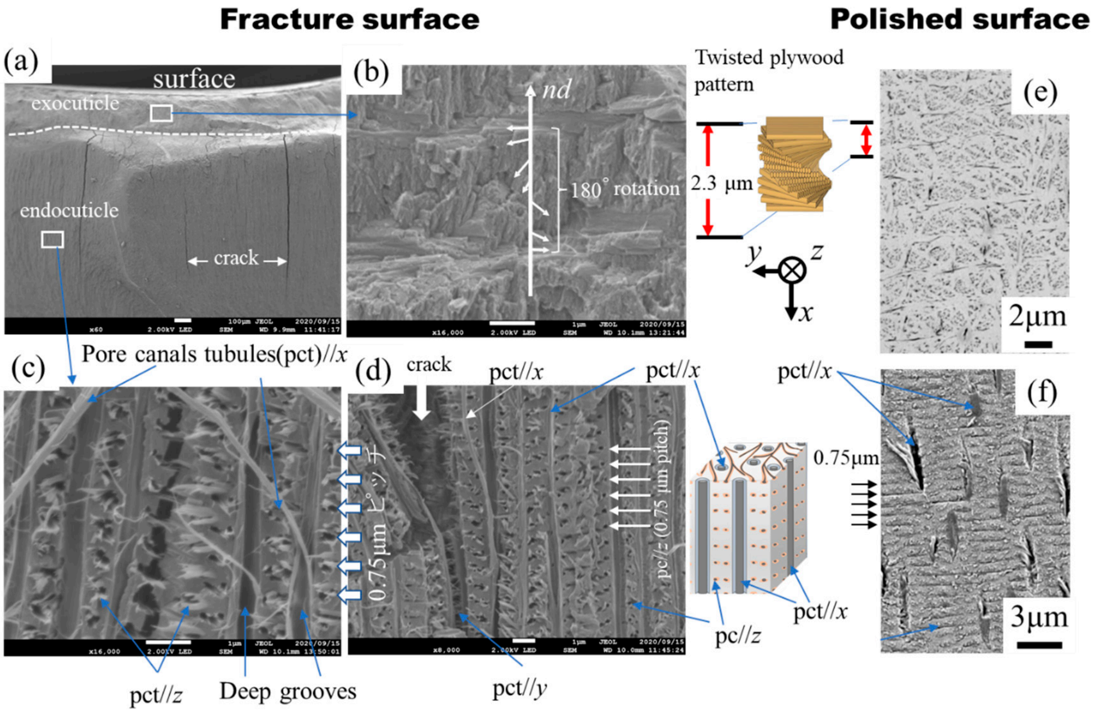

Figure S3. Comparison of SEM micrographs on (**a**) fracture surface and (**b**) polished surface in claw [1]. Here, the stacked planes gradually rotated 180° around the normal axis (*nd*) in the exocuticle of fracture surface, creating a twisted plywood structure. The plywood structure is not seen in the endocuticle.

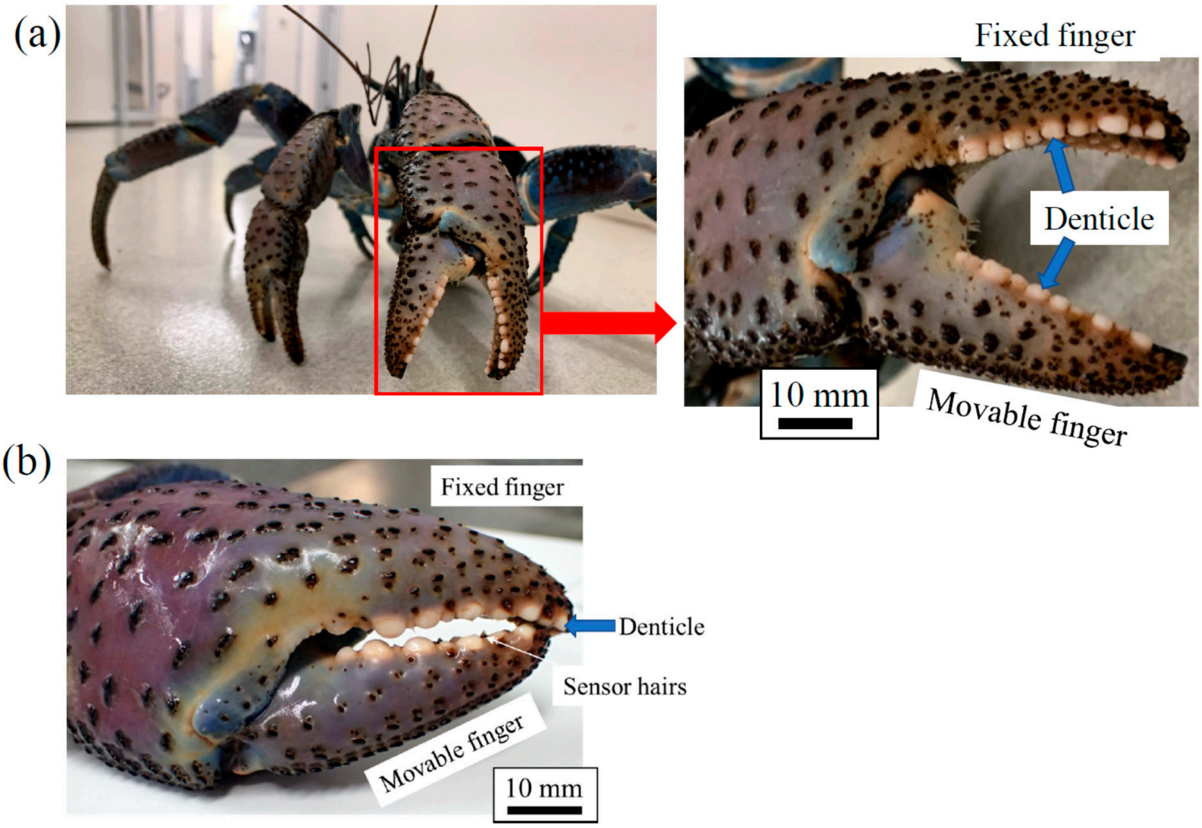

Figure S4. Heterogeneous/irregular denticles (white) of the fixed finger on the left claw of male coconut crabs; (**a**) body weight: 1,070 g, thoracic length: 62 mm [1] and (**b**) body weight: 910 g, thoracic length: 48.7 mm.

References

1. Inoue, T.; Oka, S.; Hara, T. Three-dimensional microstructure of robust claw of coconut crab, one of the largest terrestrial crustaceans, *Mater. Design* **2021,** *206*, 109765. https://doi.org/10.1016/j.matdes.2021.109765.
